# Significance of ERβ expression in different molecular subtypes of breast cancer

**DOI:** 10.1186/1746-1596-9-20

**Published:** 2014-01-23

**Authors:** Liying Guo, Jie Meng, Dilimina Yilamu, Adina Jakulin, Minggang Fu, Bowei Wang, Gulinaer Abulajiang

**Affiliations:** 1 Department of Breast, Digestive & Vascular Center, First Affiliated Hospital of Xinjiang Medical University, No. 137, South Liyushan Road, Xinjiang 830054, P. R. China; 2 Department of Pathology, First Affiliated Hospital of Xinjiang Medical University, No. 137, South Liyushan Road, Xinjiang 830054, P. R. China

**Keywords:** Breast cancer, ERβ, Molecular subtypes

## Abstract

**Purpose:**

This study is to investigate the estrogen receptor β (ERβ) expression in molecular subtypes of breast cancer and clinic significance of ERβ expression.

**Method:**

The ERβ expression was detected in 730 cases of breast cancer tissue specimens by immunohistochemistry. Twenty-one patients were censored during 2–10 years follow-up. The difference in ERβ expression was analyzed by Pearson Chi-square Test. Its correlation with estrogen receptor α (ERα), progesterone receptor (PR) and human epidermal growth factor receptor 2 (Her-2) was analyzed by Spearman rank correlation. The accumulative tumor-free survival rate was calculated by Kaplan-Meier method and difference in survival rate was analyzed by Log-rank test. Cox regression was used for multi-factor analysis.

**Result:**

The ERβ expression was significantly different among the molecular subtypes of breast cancer (P < 0.05). The ERβ expression in breast cancer was positively correlated with Her-2 (P < 0.05) while it had no correlation with ERα and Her-2. The expression of ERα was negatively correlated with Her-2 (P < 0.01) whereas positively correlated with PR (P < 0.01). The expression of PR was negatively correlated with Her-2 (P < 0.05). The tumor-free survival rate in patients with positive ERβ expression was significantly lower than that in patients with negative ERβ expression.

**Conclusion:**

Positive ERβ expression is a poor prognostic factor of breast cancer.

**Virtual slides:**

The virtual slides for this article can be found here: http://www.diagnosticpathology.diagnomx.eu/vs/1084557586106833

## Introduction

Estrogen receptor (ER) and progesterone receptor (PR) are steroid hormone receptors that belong to the nuclear receptor superfamily. Clinically, ER and PR are hormone dependent receptors of cancer cells. The human epidermal growth factor receptor 2 gene (Her-2) encodes a transmembrane receptor-like protein, which has tyrosine kinase activity. ER, PR and Her-2 play important roles in prognosis of breast cancer. There are two types of ER, which are ERα and ERβ. ERβ was cloned in 1996 by Kuiper et al. [1] from the cDNA library of rat prostate and ovary. ERα and ERβ both play important roles in regulating the biological function of the estrogen [2]. It is reported that ERβ has prognostic value in breast cancer [3,4]. For example, Jensen et al. [5] reported that ERβ expression was closely related to tumor growth and invasion of breast cancer and was a prognostic factor for breast cancer.

Based on genetic profiles of ERα, PR and Her-2, Perou et al. [6] proposed the molecular subtypes of breast cancer in 2000, which included the luminal subtype, Her-2 overexpression type, basal-like type and normal breast-like type. In 2003, Sorlie et al. [7] further divided the luminal subtype into luminal A type and luminal B type. It is well-known that molecular subtypes are closely related to breast cancer prognosis. Luminal subtype of breast cancer has better prognosis than other subtypes and luminal A subtype has the best prognosis of all molecular subtypes [8]. However, in clinical practice, expression of ER, PR and HER-2 evaluated by immunohistochemistry are used to identify various breast cancer subtypes. Evidence indicates that subtypes of breast cancer identified by DNA microarray may approximately relate to expression of commonly used markers in breast cancers: ER, PR and HER-2 status [9]. Moreover, immunohistochemistry is much easier and cheaper than gene microarray, but provides significant information to discriminate good and poor prognosis breast cancer [10-12]. Thus, we used expression of ER, PR and HER-2 to identify molecular subtypes of breast cancer in this study.

In this study, the ERβ expression was examined by immunohistochemical staining in 730 cases of breast cancer. The ERβ expression was analyzed in different molecular subtypes of breast cancer. And the correlation of ERβ with ERα, PR and Her-2 was also studied. Additionally, the accumulative tumor-free survival rate of breast cancer patients with different expression levels of ERβ was further compared. Moreover, the prognostic role of ERβ in breast cancer was evaluated by Cox regression analysis.

## Materials and methods

### Samples

Seven hundred and thirty cases of patients with pathologically confirmed breast cancers were enrolled in this study. They were all diagnosed and treated in the First Affiliated Hospital of Xinjiang Medical University from January 2000 to December 2010. They had invasive ductal carcinoma, with clinical stages of stage 0, stage I and stage II. Their clinical data were complete and were shown in Table 1. The patients were followed up for 2 to 10 years. During the follow-up time, 21 patients were censored. Patients died of other diseases, lost to follow-up at the time of last contact or before study cut-off were censored.

**Table 1:** Clinical data of breast cancer patients used in this study (n (%))

**Clinical features**	**Cases (%)**	**Clinical features**	**Cases (%)**
Menses		Staging	
Menostasis	355 (50.1)	Stage 0	218 (30.7)
Non-menostasis	354 (49.9)	Stage 1	339 (47.8)
Age (years)		Stage 2	152 (21.4)
≤ 39	164 (23.1)	Chemotherapy	
≥ 40 ~ 59	399 (56.3)	Yes	595 (83.9)
≥ 60	146 (20.6)	No	114 (16.1)
Tumor size (cm)		Radiotherapy	
≤ 2	329 (46.4)	Yes	454 (64.0)
> 2 -- ≤ 3	380 (53.6)	No	255 (36.0)
Lymph node metastasis		Endocrine therapy	
L0	385 (54.3)	Yes	290 (40.9)
L1~4	324 (45.7)	No	419 (59.1)

Prior written and informed consent was obtained from every patient and the study was approved by the ethics review board of Xinjiang Medical University.

### Immunohistochemical staining

Breast cancer tissue specimens were fixed in 10% formaldehyde for 24 h and then embedded in paraffin. Tissue specimens were sliced into 3 um sections and placed in a 70°C oven overnight. Sections were then dewaxed in xylene for 20 min and rehydrated in graded alcohols. Endogenous peroxidase was blocked by using a 3% solution of hydrogen peroxide for 10 min. For antigen retrieval, sections were placed in EDTA antigen retrieval solution and boiled for 20 min. After naturally cooling to room temperature and washing with PBS, sections were incubated with primary antibodies of polyclonal rabbit anti-human ERβ antibody (BY-02101, Shanghai Yueyan Biological Technology, CO., Ltd., Shanghai, China), monoclonal rabbit anti-human ERα antibody (ZA-0102, Beijing Zhong Shan-Golden Bridge Biological Technology CO., Ltd., Beijing, China), monoclonal rabbit anti-human PR antibody (ZA-0255, Beijing Zhong Shan-Golden Bridge Biological Technology CO., Ltd., Beijing, China) and monoclonal rabbit anti-human Her-2 antibody (4B5, Ventana Medical Systems Inc., Tuscon, Arizona, USA) at 37°C for 1 h in the dark. Then sections were incubated with secondary antibodies of HRP conjugated anti-rabbit IgG at 37°C for 30 min in the dark. After antibody incubation, sections were developed with DAB chromogenic reagent for 5 min and counterstained with haematoxylin. After hydrochloric acid differentiation and dehydration in graded alcohols, sections were mounted with neutral gum. Positive samples were used as the positive controls. In the negative controls, the secondary antibodies were replaced with PBS.

### Determination of ERβ, ERα, PR and Her-2 expression levels

The immunohistochemical staining results were evaluated by an experienced pathologist. Cells with brown staining were ERβ positive cells. Five fields at high-magnification were randomly taken. ERβ positive rate was the ratio of the number of ERβ positive cells to the total number of cells in each field. ERβ positive rate less than 1% was defined as ERβ negative (ERβ (−)). A positive rate between 1% and 10% was defined as ERβ weak positive (ERβ (+)). ERβ positive rate between 10% and 50% was defined as ERβ positive (ERβ (++)). ERβ positive rate over than 50% was ERβ strong positive (ERβ (+++)).

ERα and PR positive cells were also stained brown. According to the “Guideline Recommendations for Immunohistochemical Testing of Estrogen and Progesterone Receptors in Breast Cancer” published by American Society of Clinical Oncology (ASCO) and College of American Pathologists (CAP) in 2010, a positive staining rate of > 1% was considered positive expression.

Based on “Her-2 Detection Guide” published by Chinese Journal of Pathology in 2009, positive staining of Her-2 was defined as: 0, no staining; 1+: weak or incomplete cell membrane staining; 2+: > 10% of invasive cancer cells showing weak to moderate intensity with complete but nonuniform membrane staining or < 30% of invasive cancer cells showing strong, complete and uniform membrane staining; 3+: > 30% of invasive cancer cells showing strong, complete and uniform membrane staining.

### Statistical analysis

SPSS17.0 software was used for statistical analysis. Expression difference was analyzed by Pearson chi-square test and correlation among different expressions was assessed by Spearman’s Rank-order correlation. The accumulative tumor-free survival rate was calculated using the Kaplan-Meier method. The difference in tumor-free survival between groups with different ERβ expression was compared by Log-rank test. Analysis of multivariate prognostic factors was performed by Cox regression model. P < 0.05 was considered statistically significant.

## Results

### ERβ, ERα, PR and Her-2 expression in breast cancer

**Figure 1:**
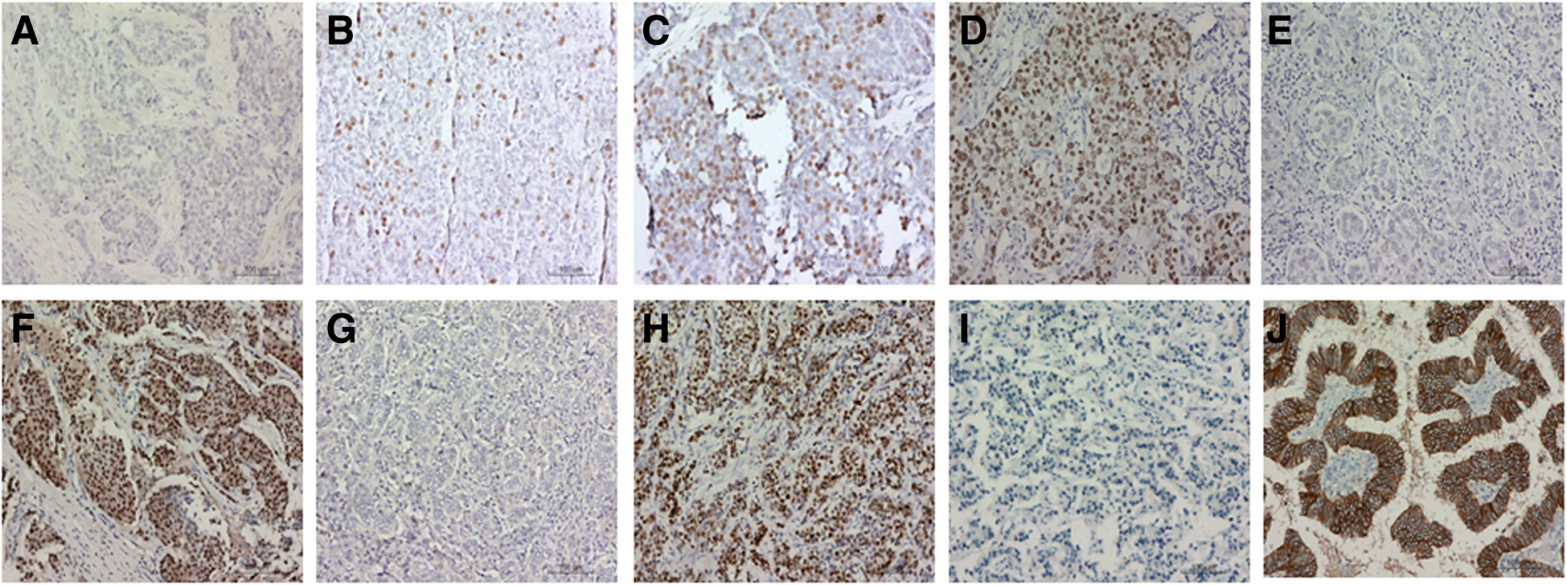
**Expression analysis of ERβ, ERα, PR and Her-2 in breast cancer tissue.** Immunohistochemistry was performed to detect ERβ, ERα, PR and Her-2 expression. Representative images were shown. Cells with brown granules were positive cells. Scale bar, 100 μm. **(A)** ERβ negative tissue (ERβ (−)). **(B)** ERβ weak positive expression tissue (ERβ (+), cancer cells with a positive rate of < 10%). **(C)** ERβ positive expression tissue (ERβ (++), cancer cells with a positive rate between 10% and 50%). **(D)** ERβ high expression tissue (ERβ (+++), cancer cells with a positive rate of > 50%). **(E)** ERα negative tissue. **(F)** ERα positive tissue. **(G)** HR negative tissue. **(H)** HR positive tissue. **(I)** Her-2 negative tissue. **(J)** Her-2 positive tissue.

To determine the expression of ERβ, ERα, PR and Her-2 in breast cancer tissue, immunohistochemical staining was performed. Representative results were shown in Figure 1. Cells with brown granules were positively stained. As described in “Materials and Methods”, ERβ expression levels were divided into ERβ (−) (Figure 1A), ERβ (+) (Figure 1B), ERβ (++) (Figure 1C) and ERβ (+++) (Figure 1D). ERα negative and positive expression was shown in Figure 1E and Figure 1F. PR negative and positive expression was shown in Figure 1G and Figure 1H. Her-2 negative and positive expression was shown in Figure 1I and Figure 1J. Molecular subtypes of breast cancer were defined based on the expression levels of ERα, PR and Her-2.

### ERβ expression is significantly different in different molecular subtypes of breast cancer

To compare the difference in ERβ expression among different molecular subtypes of breast cancer, we did Pearson chi-square test. Firstly, the molecular subtypes of 730 cases of breast cancer were determined based on the expression levels of ERα, PR and Her-2 (Table 2). A total of 545 cases of breast cancer were identified with molecular subtypes, including 202 cases (37.1%) of lumina A type (ERα+, PR+, Her-2-), 147 cases (27.0%) of luminal B type (ERα+, PR+, Her-2+), 95 cases (17.4%) of Her-2 over-expression type (ERα-, PR-, Her-2+) and 101 cases (18.5%) of basal like type (ERα-, PR-, Her-2-). Then we analyzed the expression of ERβ in different molecular subtypes. As shown in Table 2, the ERβ expression was significantly different among different molecular subtypes of breast cancer (*χ*^2^ = 20.543, P < 0.05). In cases with ERβ (−) expression, Lumina A type breast cancer had the highest proportion (34.2%, 97/284) while Her-2 over-expression type (17.3%, 49/284) and basal like type (22.2%, 63/284) had the lowest proportion. The basal like type had higher proportion of cases with ERβ (+++) expression (20.8%, 11/53) than that of cases with ERβ (+) expression (13.0%, 30/154) and ERβ (++) expression (13.0%, 7/54). Therefore, ERβ was differentially expressed in different molecular subtypes of breast cancer.

**Table 2:** Expression of ERβ in different molecular subtypes of breast cancer

	**Total cases**	**ERβ (-)**	**ERβ (+)**	**ERβ (++)**	**ERβ (+++)**	***χ***^**2**^	**P**
		**Cases (%)**	**Cases (%)**	**Cases (%)**	**Cases (%)**		
Luminal A type	202	97 (48.0)	66 (32.7)	27 (13.4)	12 (5.9)	20.543	0.015*
Luminal B type	147	75 (51.0)	38 (25.9)	11 (7.5)	23 (15.6)		
Her-2 over-expression type	95	49 (51.6)	30 (31.6)	9 (9.5)	7 (7.4)		
Basal like type	101	63 (62.4)	20 (19.8)	7 (6.9)	11 (10.9)		

Note: Pearson chi-square test, *P < 0.05.

### Correlation analysis of ERβ, ERα, PR and Her-2 expression

To analyze the correlation of ERβ, ERα, PR and Her-2 expression, Spearman’s Rank-order correlation was performed in 730 cases of breast cancer patients. The results were shown in Table 3. ERβ expression and Her-2 expression was positively correlated (P < 0.05). ERα expression and Her-2 expression was negatively correlated (P < 0.01). And, there was a negative correlation between PR expression and Her-2 expression (P < 0.05). A positive correlation was found between ERα expression and PR expression (P < 0.01). However, no correlation between ERβ expression and ERα expression or PR expression was identified (P > 0.05).

**Table 3:** Correlation analysis of ERβ, ERα, PR and Her-2 expression

	**ERβ**	**ERα**	**PR**	**Her-2**
ERβ	1	0.021	0.05	0.078*
ERα	0.021	1	0.540**	-0.101**
PR	0.05	0.540**	1	-0.086*
Her-2	0.078*	-0.101**	-0.086*	1

Note: Spearman’s Rank-order correlation, *P < 0.05, **P < 0.01.

### The tumor-free survival rate of the patients with positive expression of ERβ is significantly decreased

**Figure 2:**
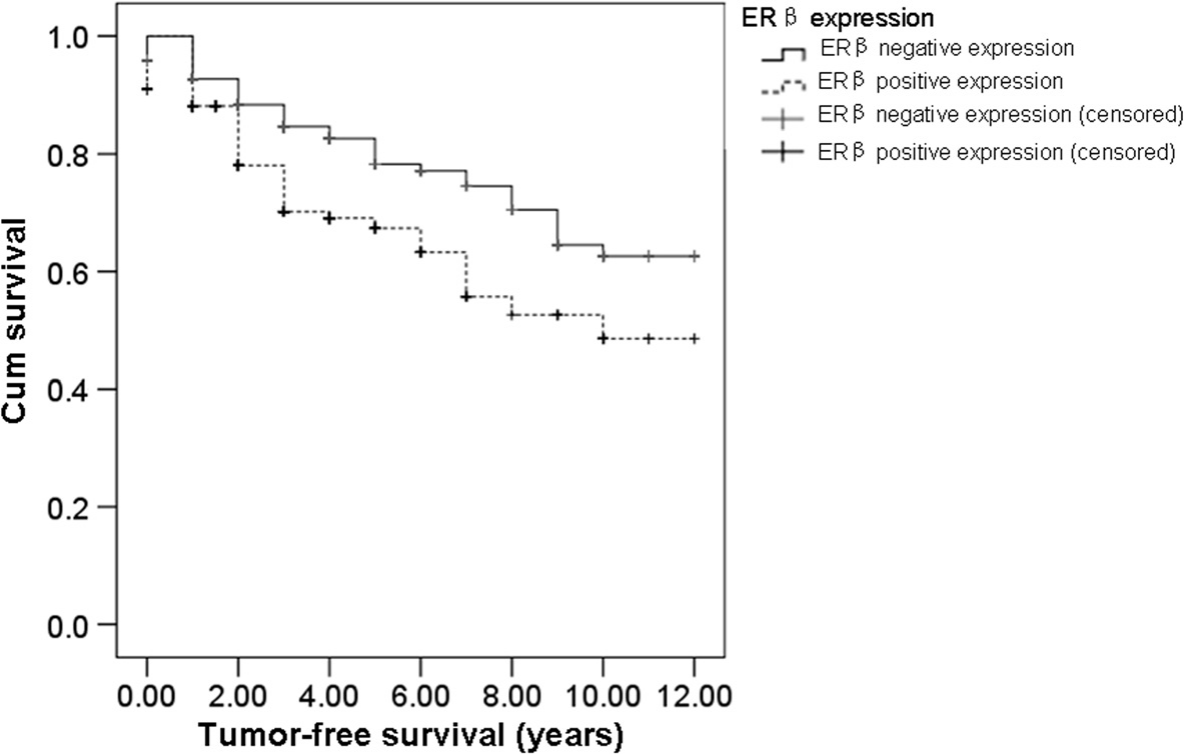
**Survival analysis of breast cancer patients with negative and positive ERβ expression.** Kaplan-Meier survival curve was displayed. The differences in survival time were analyzed by Log-Rank test. The patients (730 cases) were followed up for 2 to 10 years. During the follow-up time, 21 patients were censored. Patients died of other diseases, lost to follow-up at the time of last contact or before study cut-off were censored.

To investigate the effect of ERβ expression on survival of breast cancer patients, the accumulative tumor-free survival rate was analyzed by Kaplan-Meier method and the differences in survival time were analyzed by Log-Rank test. The survival curve of ERβ negative and positive expression patients (including ERβ (+), ERβ (++) and ERβ (+++) ) was shown in Figure 2. The median tumor-free survival rate in patients with negative ERβ expression was 9.341 years. Meanwhile, the median tumor-free survival rate in ERβ positive expression patients was 7.850 years, significantly lower than that in low ERβ expression patients (Log-rank test, *χ*^2^ = 10.748, P < 0.01). This result suggests that patients with positive ERβ expression had shorter tumor-free survival time and poor prognosis.

### Analysis of prognostic factors for breast cancer

We also analyzed the prognostic factors for breast cancer by the Cox multivariate analysis. The analyzed factors included clinical stage, ERβ expression, ERα expression, PR expression, Her-2 expression and postoperative chemotherapy, radiotherapy and endocrine therapy. As shown in Table 4, clinical stage I was a risk factor for breast cancer prognosis, with OR value = 0.164 and P = 0.000. Clinical stage II was also a risk factor for breast cancer prognosis, with OR value = 0.408 and P = 0.001. And, the OR value and P value of ERβ expression was 0.481 and 0.016, respectively, indicating that ERβ expression was a prognostic risk factor for breast cancer. In addition, postoperative chemotherapy was also a prognostic risk factor for breast cancer, with OR value = 0.334 and P = 0.001. However, ERα expression, PR expression, Her-2 expression, radiotherapy and endocrine therapy were not independent prognosis factors. Therefore, the independent prognostic risk factors for breast cancer included clinical stage, ERβ expression and postoperative chemotherapy.

**Table 4:** Analysis of prognostic factors for breast cancer by Cox multivariate analysis

**Variances**	**Regression coefficient**	**Standard error**	**Wald value**	***P *** **value**	**OR value**	**95.0% confidence intervals**	
Clinical staging			22.570	0.000*			
Stage I	-1.807	0.402	20.205	0.000*	0.164	0.075	0.361
Stage II	-0.897	0.271	10.976	0.001*	0.408	0.240	0.693
ERβ expression	-0.732	0.303	5.834	0.016*	0.481	0.266	0.871
ERα expression	0.074	0.286	0.067	0.795	1.077	0.615	1.888
PR expression	0.518	0.315	2.704	0.100	1.679	0.905	3.115
Her-2 expression	-0.108	0.242	0.199	0.656	0.898	0.559	1.442
Chemotherapy	-1.096	0.326	11.337	0.001*	0.334	0.177	0.633
Radiotherapy	-0.515	0.270	3.648	0.056	0.597	0.352	1.014
Endocrine therapy	-0.016	0.279	0.003	0.954	0.984	0.570	1.700

Note: Cox multivariate analysis, *P < 0.05.

## Discussion

Breast cancer is a common malignancy in women, with high mortality rate [13]. Identification of biomarkers for early detection and new therapeutic targets of breast cancer helps to reduce the morbidity of this frequent pathology in women. To date, several breast markers have been postulated, such as ER (ERα and ERβ), PR, Her-2, BRCA1 (breast cancer susceptibility gene) and β1 integrin [14-16]. Among them, the role of ERβ in breast cancer prognosis is still controversial. In this study, the expression of ERβ in different molecular subtypes of breast cancer was compared. Our result showed that the expression level of ERβ had significant difference (P < 0.05) in the four molecular subtypes of breast cancer. In ERβ negative expression group, the proportion of luminal A type was significantly higher than the other three subtypes while the proportion of Her-2 overexpression type and basal like type was the lowest. Due to its strong invasive ability and metastasis ability, the basal like type is an independent prognostic factor of distant metastasis [17]. In this study, the basal like type had higher proportion of cases with ERβ (+++) expression, indicating that overexpression of ERβ may suggest poor prognosis of breast cancer.

Her-2 is considered to be an oncogene that is closely related to the development of breast cancer [18]. It is involved in the regulation of cell proliferation and differentiation, and its over-expression indicates high degree of malignancy, high recurrence rate, strong invasion and metastasis and poor prognosis. In this study, ERα and Her-2 expression was significantly negatively related (P < 0.01). PR expression and Her-2 expression was negatively related (P < 0.05). ERα expression and PR expression was significantly positively related (P < 0.01). These results were consistent with previous reports [19]. Moreover, ER and PR might be associated with Her-2 signal transduction pathway [20]. ERα in combination with estrogen could inhibit the expression of Her-2. Her-2 expression is up-regulated when ERα expression is down-regulated. Chung et al. [21] also found that the Her-2 expression was directly related with ERα expression. However, the role of ERβ in breast cancer and whether it could be used as a prognosis indicator of breast cancer are still controversial. Most studies suggest that ERβ is positively correlated with epidermal growth factor receptor [22]. ERβ inhibits apoptosis of tumor cells and thus ERβ positive expression suggests poor prognosis of breast cancer. However, some studies indicate that ERβ confers a good prognosis of breast cancer [23]. In this study, ERβ and Her-2 was positively related (P < 0.05), suggesting that positive expression of ERβ may be a poor indicator of breast cancer prognosis. This result was consistent with the data reported by Huang et al. [24]. They found that positive expression of ERβ indicated poor distant disease-free survival (DDFS) rather than the overall survival time. ERβ may be related to distant metastasis of breast cancer and the overall survival time of the patients with positive ERβ expression was significantly lower than the ones with negative ERβ expression.

The cumulative tumor-free survival rate was analyzed by the Kaplan-Meier method. The cumulative tumor-free survival rate of the patients with positive ERβ expression was significantly decreased. The Cox multivariate analysis showed that ERβ expression, clinical stage and postoperative chemotherapy were independent risk factors for breast cancer prognosis. The positive ERβ expression was a high-risk prognostic factor, suggesting a poor prognosis in patients with positive ERβ expression. The underlying mechanisms of the role of ERβ in breast cancer might be related with the following two aspects. One is that through binding with ERβ, estrogen can activate G protein which rapidly inhibits c-Jun N-terminal kinase (JNK) pathway and apoptosis of breast cancer cells [25]. The other one is that ERβ could regulate the expression of related genes in the Wnt signaling pathway [26]. Thus, ERβ could regulate the cell proliferation and invasion of breast cancer. And its expression is closely related with the recurrence and metastasis of breast cancer.

In summary, ERβ was differentially expressed in different breast cancer molecular subtypes. And ERβ expression was positively correlated with Her-2 expression. The cumulative tumor-free survival time in patients with negative ERβ expression was longer than the ones with positive ERβ expression. Multivariate analysis indicates that ERβ was an independent risk factor for breast cancer prognosis. Therefore, we suppose that combined detection of ERβ and ERα would be beneficial to better assess the proliferation activity of breast cancer and to improve the accuracy of prognosis evaluation in patients with breast cancer.

## Competing interests

The authors declare that they have no competing interests.

## Authors’ contributions

All authors read and approved the final manuscript. LG conceived and designed the experiments, analyzed the data and wrote the manuscript. JM, DY and MF performed the experiments. Especially, we want to thank GA, BW and AJ. GA offered help in immunohistochemistry and BW, an experienced pathologist, did the immunohistochemical evaluation. AJ offered help in the revision of the manuscript.
